# Anime tourists traveling to Japan: pilgrimage behaviors patterns and the formation of homologous emotions

**DOI:** 10.3389/fpsyg.2025.1637114

**Published:** 2025-11-17

**Authors:** Rumin Zheng, Shuo Zhen, Anning Cai, Sihan Li, Hongyu Liu

**Affiliations:** 1 School of Tourism and Social Management, Nanjing Xiaozhuang University, Nanjing, China; 2 School of Geography and Planning, Chizhou University, Chizhou, China; 3 City Development Research Institute, National Academy for Mayors of China, Beijing, China

**Keywords:** anime tourists traveling to Japan, pilgrimage behaviors, homologous emotions, destination marketing, niche tourism

## Abstract

**Introduction:**

With the rapid expansion of the tourism market and the increasing demand for personalized travel experiences, anime-themed cultural tourism has gained significant market attention due to its distinctive characteristics. This study focuses on the pilgrimage behavior and homologous emotion mechanism of Chinese anime tourists traveling to Japan.

**Methods:**

It employs research methods including questionnaires, in-depth interviews, and participatory observation.

**Results:**

The findings reveal that anime tourists traveling to Japan exhibit strong youth-oriented demographics. Their high education levels and residence in economically developed cities provide a foundation for cultural consumption. In terms of behavioral characteristics, they display highly symbolic tourism consumption, productive social media engagement, and ritualized cultural identity expression. The formation of homologous emotions among anime tourists to Japan comprises five stages: symbolization of homologous emotions, ritualization of emotional expression, hierarchical construction of shared emotional identity, community-based communication and dynamic mechanisms of shared emotional influence. Based on these findings, recommendations include optimizing operational approaches in four key areas: cultural symbols, emotional value, industrial collaborative ecosystem, and digital dissemination-to promote sustainable anime tourism development.

**Discussion:**

This study innovatively integrates emotional geography and symbolic consumption theory. It contributes valuable insights for understanding Generation Z's cultural consumption behavior and advancing the integration of culture and tourism.

## 1 Introduction

With the rapid expansion of the tourism market, the increasing diversity of tourist demands has led to more personalized tourism product development. Anime-themed cultural tourism, tourists whose primary travel purpose is anime-related activities, featuring attractions related to anime works, has gained significant market attention due to its distinctive characteristics. The Japanese anime industry, as a core carrier of Japan's cultural soft power, has formed a global cultural consumption community through cross-border storytelling and value export. In the latest Travel & Tourism Development Index, Japan ranked as the world's leading tourism destination. Japan's anime culture is driving a global tourism boom, with fans flocking to real-life locations featured in popular anime, reshaping travel patterns (Toursim News, 2025).

This cultural phenomenon, based on virtual worlds, has reshaped public aesthetic perceptions and emotional connections while also creating a unique tourism consumption model—anime pilgrimage (seichi junrei). Originally referring to religious journeys for spiritual meaning, “pilgrimage” in the anime context now describes fans visiting real-world locations featured in their favorite works; these places are called “holy sites.” These sites can range from vibrant city streets to ordinary spaces that gain attention due to their connection to anime (James Taylor, 2023). Anime tourists engage in pilgrimage by visiting filming locations, participating in anime-themed events, and purchasing anime merchandise, reconstructing the emotional value of fictional stories in real-world spaces.

The strong emotional projection tied to anime characters and real locations is a key feature distinguishing anime tourism from other cultural and film-induced tourism. This shift from “sightseeing” to “pilgrimage” reflects deeper psychological engagement. Due to lingering health and safety concerns, Chinese tourists have shown a preference for short-haul destinations, with Japan being a top choice due to proximity and perceived safety. Chinese tourists are increasingly drawn to Japan's rich cultural offerings, from traditional tea ceremonies to local festivals, reflecting a growing interest in immersive travel experiences. Aligning with global trends, Chinese tourists are also more conscious of their travel's environmental and social impacts, seeking sustainable tourism options, eco-friendly accommodations, and experiences that support local culture without causing overtourism (Oliver, 2024).

China is the largest source of tourists for Japan's tourism industry. It boasts a massive audience for anime culture, making it one of the world's largest markets for anime consumption. China's pan-anime user base exceeds 400 million people, with strong purchasing power. Many Japanese anime works have accompanied the growth of China's Generation Z (born between 1995 and 2009). These animated productions carry deep emotional connections with this demographic, forming what we call homologous emotions, which also underscores the unique emotional bond between Chinese and Japanese anime culture. Despite both belonging to the East Asian cultural sphere, Japanese anime's distinct aesthetics still hold a heterogeneous appeal for Chinese tourists. Additionally, young Chinese tourists are known for their highly active social media sharing and “check-in”-style pilgrimages. These factors provide a real-world foundation for exploring the emotional interaction mechanisms between screen culture (virtual consumption) and on-site experiences (physical tourism).

On January 8, 2019, Mafengwo (a Chinese travel platform) and the Japan Anime Tourism Association announced a strategic partnership to provide Chinese tourists with comprehensive and accurate “pilgrimage” travel information, enhancing the experience (China National Radio, 2019). Since then, anime tourism has grown in importance for Japan-bound travel. By 2030, Japan's anime market is expected to reach ~48.3 billion (Precedence Research, 2021). In 2024, in bound tourism spending in Japan hit 52.29 billion, with visitor numbers reaching 36.86 million, both setting new records. This indicates the market is expanding beyond niche anime circles into broader cultural consumption. Behind this phenomenon lies a generational shift in tourism paradigms. Given this sustainable and promising market, this study focuses on Chinese anime tourists in Japan, analyzing their behaviors and motivations to provide insights for cultural tourism research and marketing. It also offers a development model—“anime devotion + local revitalization”—that other countries could adopt.

## 2 Literature review

### 2.1 Film-induced tourism in cross-border cultural tourism

Cross-border cultural tourism is a key driver of global tourism growth. Cultural elements in this sector stem from films, religious pilgrimages, heritage attractions, and festivals (Gedecho et al., 2024; Gkritzali, 2024; He et al., 2025). Among these, film-induced tourism, linking fictional narratives with real-world travel, has become a significant facilitator. Research shows that films stimulate cross-border travel by symbolizing locations, creating emotional connections, and spreading through media, resulting in “space consumption” effects (Huerta-Viso et al., 2024; Kim et al., in press).

Some scholars argue that tourists engage in symbolic consumption—films transform destinations into cultural symbols that tourists consume to affirm identity (Nieto-Ferrando et al., 2024). For example, The Lord of the Rings turned New Zealand into “Middle-earth,” with fans visiting “Hobbiton” to experience the film's world (Croy, 2004). Others suggest films create emotional attachment through place identity and imagined geographies (Ng and Chan, 2019). For instance, the Korean drama Winter Sonata portrayed Gangwon Province as a “romantic snowland,” attracting Japanese female tourists who sought out filming locations, embodying their connection to the fictional setting (Choi, 2006). Additional studies highlight how films evoke emotional projection through narrative empathy and emotional geography, motivating travel (Engelbrecht, 2022). Roman Holiday turned the Spanish Steps into a “love pilgrimage site,” where visitors relive the film's romance (Wright-Rios and Martinez-Don, 2024). With the rise of the metaverse and short videos, digital media is altering cross-border cultural experiences (Li et al., 2024). Platforms like TikTok and vlogs shorten cultural distance through visual storytelling, encouraging “online inspiration–offline visitation” behavior (Guo and Qiu, 2025). This trend's impact on travel decisions requires further study.

Anime tourism combines core features of cultural tourism—long-term IP development, immersive experiences, transmedia storytelling, and strong fan engagement—while avoiding the short-lived hype common in film tourism (Ono et al., 2020). Compared to Western cultural tourism, Japan's anime tourism benefits from geographic proximity, cultural familiarity, and relaxed visa policies, creating steady demand among Chinese tourists. Locations like Slam Dunk's Kamakura remain top destinations. While still a niche market, licensed anime tourism routes and fan-driven content are creating a sustainable cultural tourism model, surpassing some traditional film tourism destinations in longevity (Li et al., 2023). Despite anime tourism's steady share in China's outbound market, research on anime tourists remains limited, especially regarding how social media behavior influences real-world travel.

### 2.2 Pilgrimage behavior in anime tourists traveling to Japan

“Seichi junrei” (pilgrimage) originally referred to religious visits to sacred sites. In otaku culture, it describes fans traveling to real-world locations featured in anime (Seaton and Yamamura, 2017). This phenomenon has grown with the globalization of Japan's content industry (Seaton and Yamamura, 2017). Unlike regular tourism, pilgrimage emphasizes the “connection between fiction and real space.” “Holy sites” are often real locations that gain cultural meaning through their association with anime, becoming spaces where fans express emotional connections to the story (Seaton and Yamamura, 2015).

Pilgrimage is marked by strong narrative identification, consisting of four dimensions: landscape, utopian, community, and self-identity. First, landscape identity involves tourists verifying the accuracy of locations against anime depictions while exploring local culture (Nickerson, 2024). Second, utopian identity allows fans to immerse themselves in an idealized fictional world (Reijnders, 2011). Community identity builds connections among fans and locals through shared emotional experiences (Tang et al., 2024). Self-identity reflects tourists' freedom to express themselves authentically beyond daily life (Keene, 2021; Zhou, 2023).

Anime tourists traveling to Japan express their strong connection to anime through various behaviors. First, they want to visit “the world where the story takes place” and verify how closely real locations match the anime settings. Many fans also recreate scenes by copying poses or compositions from the anime, or even cosplay to bring iconic moments to life (Yang et al., 2024). This is not just a personal experience but also a way to engage with fan communities and symbolic consumption (Kim et al., 2023). Tourists often buy and collect anime merchandise exclusive to these pilgrimage sites (Yang, 2024). Additionally, they share their experiences on social media (e.g., Twitter, Bilibili, Xiaohongshu), creating a “digital pilgrimage chain” that strengthens their status within fan groups (Blackman, 2023). On a deeper level, real locations gain cultural significance beyond their geographical meaning due to the “presence” of anime characters, allowing tourists to project their emotions onto these places (Gu and Deng, 2024).

Anime pilgrimage also revitalizes local economies and reshapes cultural identities by providing steady visitor flows (Editor of Website, 2023). While pilgrimage theory explains anime tourism behavior, most studies focus on Western contexts, neglecting East Asian and Gen Z travelers (Zhang et al., 2023). More empirical research is needed on cultural barriers and synergies in anime tourism.

### 2.3 Homologous emotions in anime pilgrimage

Shared Affection refers to the common emotional connection experienced by anime pilgrimage tourists due to the same anime or film (Fiske, 1992). Since these emotions stem from the same fictional work, they tend to be similar—such as lingering regret over a character's fate, nostalgia for youthful romance, longing for an unreachable past, attachment to a fictional version of oneself, or hope for an idealized future (Leshner et al., 2025). This shared affection shapes anime tourism, linking people and places through actions like sharing photos, cosplay, and buying merchandise (Leung and Cho, 2024).

Studies show that in Japanese anime pilgrimages, shared affection forms through three blended dimensions: real, fictional, and digital spaces (Okamoto, 2015). In the real world, tourists turn physical locations into anime symbols via activities like “itasha” (decorated cars) and figure photography (Yang et al., 2024). In the fictional world, these real-world symbols strengthen emotional bonds with characters by reinforcing the imagined narrative (Shi et al., 2024). In the digital space, sharing pilgrimage videos and travel guides creates an “imagined community,” deepening emotional attachment to the story (Kim et al., 2025).

Another theory suggests shared affection arises through film sanctification (elevating the story's meaning) and ritualized tourism behaviors. Film sanctification involves naming real locations after anime scenes, visually matching them, and emotionally immersing oneself—like admiring a director's style, empathizing with characters, or idealizing the fictional world (Tang et al., 2024). These feelings manifest outwardly as character cosplay, scene reenactments, and themed festivals. Meanwhile, ritualized tourism involves standardized behaviors—pre-planned routes, posed photos to “fix” fictional memories, and wearing anime outfits to relive the story (Reichenberger, 2021).

Research also highlights how cultural tourism policies shape shared affection (Longo, 2024). Tourists prefer destinations deeply integrated with anime culture. Japan's “Anime IP + Local Identity” model, blending anime with regional traditions, fosters this connection (Wu and Lai, 2025). Government and industry efforts—like Shigeru Mizuki Road (a street in Sakaiminato featuring bronze statues of his GeGeGe no Kitaro characters)—merge tourism with fandom, attracting 1.5 million visitors in 2023 (Anime Tourism: A Global Phenomenon from Japan). Such policies guide pilgrim preferences and reinforce shared emotions.

### 2.4 Research objectives

Existing studies have provided relatively in-depth analyses of the characteristics and formation mechanisms of homologous emotions in anime tourism. However, much of this research remains descriptive, focusing primarily on observed phenomena rather than the underlying channels that connect pilgrimage behaviors with these shared emotions. A deeper investigation into this linkage is necessary to offer stronger theoretical foundations for cultural tourism research and marketing strategies.

Building upon the literature review, we selected Chinese anime tourists traveling to Japan as our research subjects and conducted surveys and interviews to address the following key questions:

What are the behavioral characteristics of Chinese anime tourists engaged in pilgrimage activities in Japan?

How do homologous emotions influence the behavior of these tourists?

What tourism marketing strategies can be developed based on the behavioral patterns and motivational mechanisms of anime pilgrimage tourists?

By answering these questions, this study aims to systematically explain the behavioral traits and emotional mechanisms of anime tourists, enriching existing research on the affective dimensions of cultural tourism. The findings will contribute theoretically by offering new perspectives on anime-themed cultural consumption, and practically by proposing strategies for innovative marketing models and optimized industry operations in cultural tourism.

## 3 Materials and methods

### 3.1 Methods design

**Figure 1:**
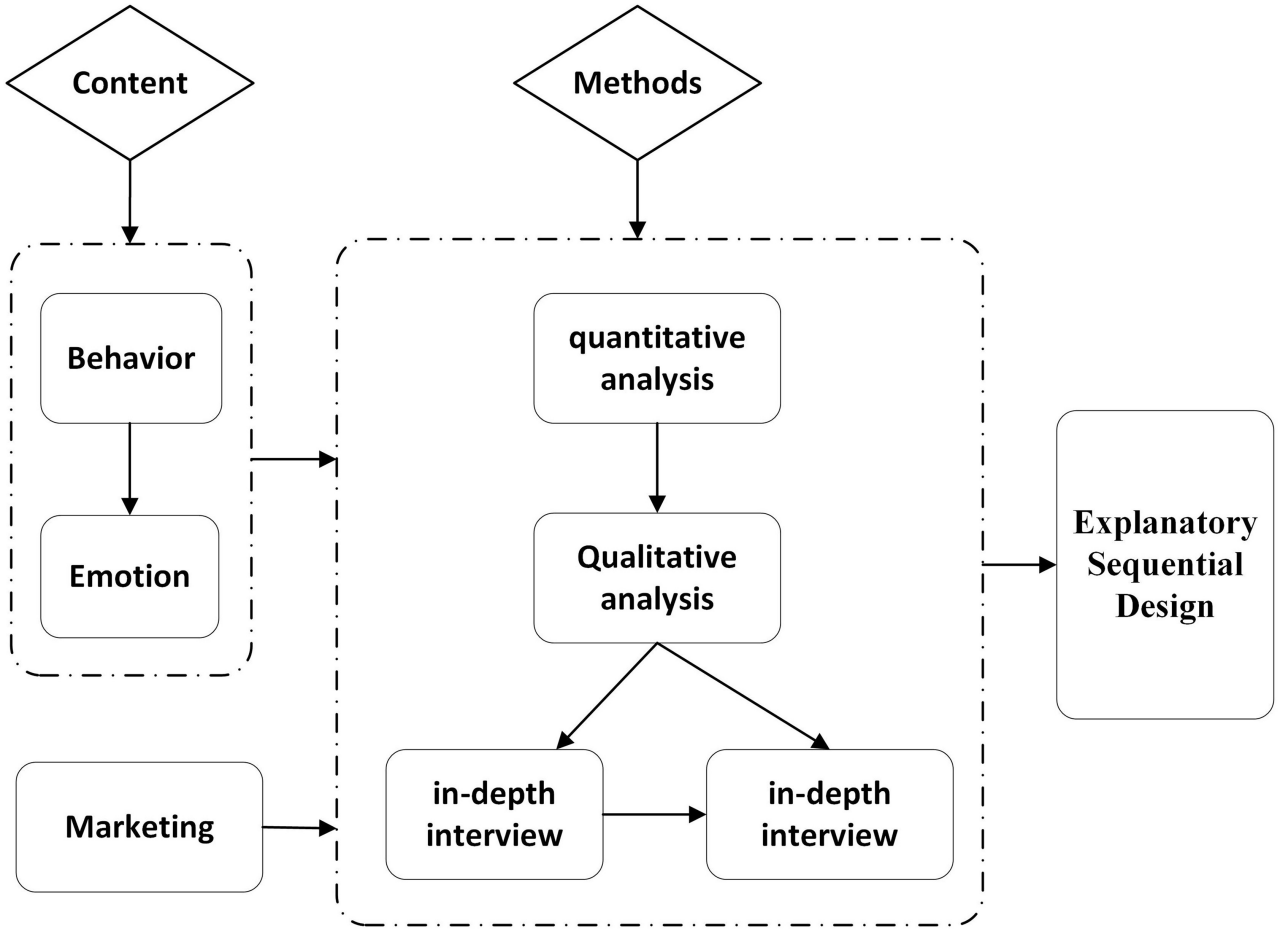
” The study framework.

To achieve the synergistic effect of quantitatively identifying phenomena and qualitatively explaining essence, this paper adopts the Explanatory Sequential Design to establish the methodological framework. In the Explanatory Sequential Design, qualitative results are primarily used to interpret and expand upon quantitative data. Quantitative data provide preliminary statistical findings, while qualitative data, gathered through in-depth interviews, help researchers understand the underlying reasons and mechanisms behind these results. This is a significant type of research design in mixed-methods research. It involves the collection, analysis, and interpretation of both quantitative and qualitative data to gain a deeper understanding of surface phenomena, root causes, and motivations related to the research problem. The study framework is presented in Figure 1.

### 3.2 Research content design

Based on the research framework, the detailed steps and methods are as follows.

#### 3.2.1 Quantitative research

Quantitative research were conducted to capture the basic information of anime tourists and to understand the explicit characteristics of their tourism pilgrimage behaviors. This part was primarily achieved through the distribution of surveys. The structured questionnaire covers indicators from five aspects drawing on relevant research literature combing several existing scales.

I. Basic information of anime tourists. This aspect includes age, gender, occupation, income, and education level. This serves as the foundation of tourism pilgrimage research (Liu et al., 2021).
II. Anime engagement behaviors. This aspect includes primary ways of accessing anime, time spent, types of anime consumed, level of participation in anime-related activities, and frequency of engagement. It reflects tourists' familiarity with anime culture and their modes of participation, providing a basis for screening typical anime tourists (Sabre, 2017; Okamoto, 2015).
III. Characteristics of tourism behaviors. This aspect includes general tourism motivation, anime tourism motivation, expenditure areas, and the specific activities in anime filming locations. This aspects draws on an existing model called the “Anime Tourism Motivation Scale,” which categorizes anime tourism motivation into five dimensions: seeking anime authenticity, novelty, escape/relaxation, socialization, and anime cultural exploration (Sabre, 2017). This model provides a systematic theoretical basis for understanding the motivations of anime tourists.
IV. Emotional experiences. This aspect includes the perceived sources of attraction, desired emotional experiences, and ways of expressing emotions among anime tourists. This section builds on research on emotional experiences in film tourism about tourists' emotional places and emotional experiences during cultural travel (Kim, 2012; Liu et al., 2021).
V. Preferences of social media use. This aspect includes the types of social media, content shared, and the degree to which social media influences tourism decision-making. This study references a research framework from a study on Turkish anime audiences and their perception of Japanese culture, particularly regarding how social media influences travel decisions (Basaran and Sunnetcioglu, 2021).

The questionnaire underwent a pretest before distribution. The test subjects were individuals in anime forums who had engaged in anime pilgrimage activities. They were contacted and completed the questionnaire through private messages on online platforms. A total of 32 valid responses were obtained. Subsequently, validity and reliability tests were conducted on the main sections of the pilot questionnaire excluding basic personal information.

Content validity of the original scale was assessed using expert judgment. The scale was developed based on existing literature on anime tourism, combined with in-depth interviews with academic experts and anime tourists, and refined through feedback. As such, due to the rigorous and standardized development process, the scale exhibits reasonable reliability in content.

Construct validity was also examined (Table A1 and Table 1). The results showed that exploratory factor analysis (EFA) extracted four factors (KMO = 0.78, cumulative variance explained = 73%), with factor loadings above 0.6 for all items, reaching a significant level. On one hand, the average variance extracted (AVE) for each factor exceeded 0.6, indicating strong convergent validity. On the other hand, the square roots of the AVE for all factors were greater than their correlation coefficients with other factors, demonstrating good discriminant validity.

**Table 1:** Cronbach's α, CR values, and AVE square roots with correlation coefficients.

**Category**	**Factor 1**	**Factor 2**	**Factor 3**	**Factor 4**
Factor 1	0.800			
Factor 2	0.635^***^	0.811		
Factor 3	0.404^***^	0.453^***^	0.810	
Factor 4	0.512^***^	0.613^***^	0.469^***^	0.805
Cronbach's α	0.713	0.738	0.736	0.702
CR	0.842	0.852	0.815	0.841

^***^p <0.01; Diagonal values represent the square roots of the AVE.

The reliability tests analysis showed that the overall Cronbach's α value of the general model was 0.832. The Cronbach's α values of four factors were all greater than 0.7. The composite reliability (CR) values of the four factors were all greater than 0.8, indicating that the scale has good internal consistency.

#### 3.2.2 Qualitative research

The qualitative research in this paper included in-depth interviews and field investigations.

Firstly, in-depth interviews conducted with 18 active anime tourists traveling to Japan, focusing on their history of anime engagement, pilgrimage experience processes, and emotional expressions. The interviews explored how tourists establish emotional connections between the virtual and the real through scene reconstruction, as well as the role of symbolic consumption in identity construction. The interview process was recorded via audio and textual documentation to ensure the full display of respondents' opinions and suggestions. Interview Outline is as following.

I. Anime tourism behavior
What types of anime merchandise do you prioritize purchasing?
How do factors influence your choices?
Do you believe purchasing souvenirs from pilgrimage sites serves as emotional attachment or social display? Could you provide an example?
What immersive anime-themed experiences have you participated in? How do these services meet your expectations of bringing screen worlds to life?
Are you willing to pay a premium for experiences with high anime fidelity? Please compare a “worthwhile” and a “disappointing” experience.
II. Anime emotional experiences
What inspired your first decision for anime pilgrimage?
Could you describe how a specific anime scene/character sparked your travel motivation?
How do you understand the relationship between anime scenes and real-life locations?
What detailed emotional experiences does the pilgrimage behavior bring you?
In the pilgrimage, what's your behavior to strengthen your emotional connection with the anime? After multiple pilgrimages, has your emotional need changed?
III. Social media and community interaction
Through which channels do you obtain pilgrimage information?
How much does user-generated content influence your itinerary planning?
How do you design and post social media content during your pilgrimage? Do you aim to replicate original scenes or incorporate personal creativity?
Have you joined any online or offline anime pilgrimage communities?
How does community interaction enhance your sense of participation?
How do you perceive “identity labels” within the community? How do these labels influence your consumption or social behavior?
IV. Construction in anime tourism
Have you attempted to interpret Japanese cultural symbols in anime?
How has this interpretation altered your perception of pilgrimage sites?
When facing cultural barriers, how do you compensate?
Do you view anime pilgrimage as a declaration of subcultural identity?
Does this identity remain hidden or become prominent in daily life and other travels?
What factors diminish or enhance your pilgrimage satisfaction?

Secondly, based on the quantitative survey and in-depth interview outline, the second author conducted field research in Japan for the anime tourism study. To mitigate potential bias from the researcher's personal experience, three measures were taken. First, during the analysis phase, preconceptions were suspended. Observations and experiences were documented in written form, separating observational records from personal opinions. Second, participant and analyst validation was employed. Research subjects and other authors were invited to verify the direction and accuracy of the researcher's experiential expressions. Finally, textual data from the field research were treated as part of the in-depth interviews and coded alongside data from other interview participants to test inter-coder reliability and thematic classification consistency.

### 3.3 Data collection

Regarding the questionnaire survey method, a total of 200 questionnaires were distributed both online and offline, with 196 collected (98% return rate). After screening and eliminating invalid responses, 189 valid questionnaires remained, yielding an effective rate of 96.4%. Online distribution was conducted through anime forums and QQ/WeChat groups, while offline distribution utilized convenience sampling at anime conventions and pilgrimage hotspots in Japan to ensure data authenticity and representativeness.

For the in-depth interviews, a purposive sampling strategy was adopted to capture a range of perspectives. The interviewees were selected from respondents in the quantitative survey who exhibited active anime engagement behaviors. Based on the questionnaire findings, this study conducted in-depth interviews with 18 active anime tourists traveling to Japan, focusing on their history of anime engagement, pilgrimage experiences, and emotional expressions. The interviews explored how tourists establish emotional connections between virtual and real worlds through scene reconstruction, as well as the role of symbolic consumption in identity construction. The entire interview process was recorded via audio and transcribed into text to ensure comprehensive documentation of respondents' opinions and suggestions.

In February 2025, the researcher conducted participatory observations in prominent anime tourism destinations, including Akihabara in Tokyo and Washimiya Town in Saitama Prefecture. Detailed records were made of tourists' pilgrimage routes, consumption behaviors, and community interactions. Observations focused on how tourists took photos, checked in at sites, engaged in cosplay, selected merchandise, and participated in other related activities. These observations, combined with the researcher's firsthand experiences, provided deeper insights into tourists' shared emotional expressions.

### 3.4 Data analysis methods

Descriptive statistics presents the demographic characteristics, consumption amounts, and other fundamental data distributions of anime pilgrimage tourists through frequency distributions and mean calculations.

Qualitative analysis strictly adheres to the three-level coding process of grounded theory. NVivo 14 software was utilized. In data preparation and initial coding the transcripts of in-depth interviews, field observation notes, and social media texts referenced by interviewees were meticulously transcribed to create a textual database. In the axial coding stage of grounded theory, logical coherence was demonstrated through a structured relational model, systematically integrating causal chains, processual relationships, and conditional associations derived from the open-coded concepts.

In selective coding stage, core categories were refined from axial coding to establish the study's logical framework.

Validity measures are used. Triangulation was employed to cross-validate interview transcripts, observational data, and online textual materials. Two tourism experts were invited for peer review, and a complete audit trail—including raw data and coding memos—were maintained for verification. Inter-coder reliability between two authors reached 82%. Theoretical saturation was tested and sampling ceased when additional data no longer generated new concepts, confirming model saturation.

### 3.5 Ethical approval

This study received approval from the ethics review board of the author's institution. All participants provided written informed consent prior to inclusion, with clear explanations of the research purpose, process, potential risks, and the right to withdraw, ensuring voluntary participation. Data underwent strict anonymization: real names were replaced with ID codes, and identifiable information was removed. Only the paper's authors had access to raw data on a secure server to safeguard participant privacy.

## 4 Results

### 4.1 Basic characteristics of anime tourists traveling to Japan

The basic characteristics of anime tourists traveling to Japan is shown in Table 2. In terms of demographic characteristics, anime tourists traveling to Japan exhibit a notably young demographic, with “Post-95s” and “Post-00s” forming the majority, accounting for 90% of the total. These Generation Z tourists grew up during the global spread of Japanese anime, deeply engaging with Japanese culture through anime and developing a “pilgrimage complex.” In terms of gender distribution, female tourists outnumber male tourists, which may be closely related to the widespread popularity of female-oriented anime works in Japanese anime.

**Table 2:** The basic characteristics of anime tourists traveling to Japan.

**Categories**	**Items**	**Rate**
Age	18–25	68%
	26–30	22%
	31–40	10%
Gender	Male	64%
	Female	36%
Occupation	Students	42%
	Office workers	34%
	Freelancers	18%
	Other	6%
Education	Master's degree or higher	18%
	College/Bachelor's degree	76%
	High school or below	6%
Residence	Economically developed cities	91%
	Less developed cities	9%
Monthly income level (non-students) (RMB)	<5,000	11%
	5,000–10,000	43%
	10,000–20,000	32%
	>20,000	16%
Monthly parental support (Students)	<2,500	14%
	2,500–5,000	67%
	>5,000	19%
Spending on Japan trip	<5,000	19%
	5,000–10,000	47%
	10,000–20,000	21%
	>20,000	13%

Regarding social characteristics, occupational data indicate that anime tourists traveling to Japan are primarily students, young professionals, and freelancers. Due to their flexible schedules and consumption preferences, these groups possess strong cultural consumption capabilities and information acquisition abilities, making them the main force in anime tourism. Additionally, anime tourists traveling to Japan generally have higher education levels, with only 6% holding a high school diploma or below. As anime IPs often integrate elements of history, philosophy, and science fiction, highly educated tourists are more likely to understand and resonate with them.

In terms of economic characteristics, anime tourists traveling to Japan mainly come from China's developed cities, such as Beijing, Shanghai, Nanjing, Guangzhou, and Xiamen. Those from less-developed cities account for <10%. Higher income levels, an open attitude toward cultural consumption, and policy convenience in developed cities provide the foundation for anime tourists traveling to Japan. Moreover, anime tourists traveling to Japan tend to have higher personal incomes or receive substantial financial support from their parents. The relatively high economic cost of traveling to Japan and Japan's economic screening policies for tourism objectively select for highly educated groups with greater economic capacity and consumption willingness to undertake such non-essential cultural expenditures.

### 4.2 Behavioral characteristics of anime tourists traveling to Japan

The behavioral characteristics of anime tourists traveling to Japan differ significantly from those of ordinary sightseers or general cultural tourists. This study examines their behavioral characteristics from both quantitative (Table 3) and qualitative perspectives (Table 4). They exhibit unique behavioral traits, which can be categorized into the following three aspects.

**Table 3:** Behavioral characteristics of anime tourists visiting Japan.

**Category**	**Specific behaviors**	**Percentage**	**Related elements**	**Percentage**
Cultural tourism consumption	Photo-taking at landmarks	86	Anime merchandise	68
	Collecting limited-edition goods	68	Themed dining & lodging	52
	Cosplay (role-playing)	54	Attraction tickets	46
Social media engagement	Comparing photos	70	WeChat/Weibo	74
	Posting travel guides & tips	64	Instagram/Twitter	62
	Video recordings	52	Xiaohongshu (RED)	58
			TikTok	42
Cultural identity & emotional connection	Nostalgia & personal memories	72	Storyline resonance	76
	Identity affirmation	66	Character design	64
	Emotional engagement	62	Artistic style	58

**Table 4:** Behavioral characteristics of anime tourists traveling to Japan by ground theory.

**Selective coding**	**Axial coding**	**Open coding**
Symbolization of cultural tourism consumption	Check-in behavior	Visiting multiple sacred sites in a tight schedule Spending hours at specific locations for detailed, immersive experiences
	Scarcity-driven purchases	Limited-edition figurines, artbooks, character merchandise Themed café role-playing, anime-themed hotel stays, and other immersive services
	Social media showcasing	Taking photos and posting them on social platforms Sharing with anime fan communities Sharing with online friends
	Value appreciation	Collectible anime derivatives with cultural value Translating the cultural value of anime ip into premium consumption
Productive nature of social media behavior	Content creation phase	Posting photos of anime filming locations Sharing comparison shots between real-life sites and anime scenes Uploading cosplay videos Posting photos with anime characters Creating derivative works by blending anime clips and real-life locations
	Content-sharing phase	Exchanging travel guides with friends on social media Sharing travel tips within anime communities Fragmentarily posting travel advice on online forums
	Content dissemination phase	Commenting on trending videos/posts to maintain their visibility Gaining widespread influence after sharing self-generated videos Becoming a hot topic and attracting inquiries from others Receiving financial sponsorships and further increasing engagement
Homologous expression of cultural identity	External manifestations	Ritualization as sacred and standardized acts Posing in alignment with characters Replicating costumes Re-enacting scenes Focusing on finely detailed settings Mimicking character behaviors, outfits, and props
	Internal logic	Visiting anime locations and engaging with character imagery to express aesthetic appreciation Role-playing to affirm an identity aligned with anime content Purchasing anime merchandise to demonstrate community belonging Chasing limited-edition items as an appreciation of *mono no aware* (the beauty of transience) Seeking digital-era identity recognition through social media documentation and expression

#### 4.2.1 Symbolization of cultural tourism consumption

The cultural tourism consumption of anime tourists is highly symbolic. On one hand, product-based consumption focuses on limited-edition figurines, art books, character merchandise, and other collectible anime derivatives, forming a closed-loop process of scarcity-driven purchases → social media showcasing → value appreciation. On the other hand, service-based consumption favors immersive experiences such as cosplay in themed cafés or stays in anime-themed hotels. Both types of consumption are accompanied by photo-taking and “check-in” behaviors, through which tourists physically engage with anime to reinforce emotional connections. Additionally, they often purchase corresponding anime merchandise near filming locations.

Anime tourists traveling to Japan exhibit fragmented yet in-depth time allocation in their consumption behavior. They often densely visit multiple pilgrimage sites within a limited schedule while spending several hours at specific locations for detailed experiences. Furthermore, the cultural value of anime IPs translates directly into consumption premiums. These tourists are willing to spend more on limited-edition merchandise or immersive cultural experiences such as themed dining, accommodations, and visits to anime filming locations. For example, homestays at settings featured in Your Name. command prices several times higher than non-anime-related areas yet maintain high occupancy rates.

#### 4.2.2 Productive nature of social media behavior

Social media plays a critical role in anime tourism by facilitating content creation, sharing, and dissemination. This includes comparison photos, travel guides, and video logs. Major platforms used are WeChat, Weibo, Instagram, Twitter, and Xiaohongshu.

During the content creation phase, anime tourists traveling to Japan post comparison photos of pilgrimage sites and cosplay videos. These actions form an emotional loop linking anime to reality and back again. In the content-sharing phase, organized online communities—such as forums and fan groups—play a vital role in exchanging travel tips and coordinating group pilgrimages, fostering a sense of cultural unity. In the content dissemination phase, high-quality posts go viral due to the network effect, attracting massive engagement from fellow enthusiasts. For instance, a popular TikTok vlog documenting a pilgrimage site can garner millions of views, directly boosting inbound anime tourism traffic.

Through these social media behaviors, anime tourists traveling to Japan construct dual identities that bridge the virtual and real worlds. Additionally, they reinterpret and expand anime narratives by creating fan fiction and derivative works, which are rapidly shared and amplified via social platforms. Such fan-driven content enriches character depth, fulfills unaddressed storylines, and draws more enthusiasts into the fandom.

#### 4.2.3 Homologous expression of cultural identity

The cultural identity behaviors of anime tourists traveling to Japan are deeply rooted in anime, often assuming a sacred, ritualistic quality. By reenacting scenes—through aligned poses, replicated outfits, or meticulous location photography—they mirror the actions of anime characters, thereby expressing affinity for the plot, artistry, and ethos of the works.

Externally, this homologous expression manifests in standardized “pilgrimage” rituals (originally a religious quest for spiritual meaning, here referring to fans' on-site reenactments). Cosplayers, for example, obsessively replicate character costumes, props, makeup, and mannerisms to achieve authentic immersion. Any deviation risks being labeled OOC (out of character).

Internally, these acts—whether scene reenactments or cosplay—serve as both aesthetic statements and identity markers. A preference for “limited-edition” items further intensifies regional belonging and fan-cultural solidarity. This practice blends Japan's traditional mono no aware aesthetic with digital-era community dynamics, forging a distinct subcultural model.

### 4.3 Formation mechanism of homologous emotions in anime tourism

Research findings indicate that ~84% of respondents cited “wanting to experience anime settings” as their primary travel motivation. This often stems from deep empathy with specific anime scenes, works, or characters—referred to as homologous emotions. These emotions drive anime enthusiasts to visit filming locations in person, participate in themed events, and project their virtual emotions into the real world through immersive cultural experiences. The formation of this homologous emotional drive is summarized in Table 5 and unfolds across five stages.

**Table 5:** Results of the formation of the homologous emotional drive by ground theory.

**Selective coding**	**Axial coding**	**Open coding**
Symbolization of homologous emotions	Visual symbol recognition for emotional perception	Identifying iconic scenes in anime Establishing standardized visual symbolic features Activating memories associated with anime symbols
	Cultural meaning decoding for emotional cognition	Interpreting symbolic meanings within complex cultural networks Transforming ordinary geographical spaces into real-world representations of anime scenes Discovering anime-specific settings and behaviors in real-life locations Imbuing physical spaces with specific emotional and cultural significance
	Personalized meaning reconstruction for emotional internalization	Creatively modifying anime expressions to convey individual perspectives Filming fan-made short videos Writing travelogues about pilgrimage trips Designing custom route maps and other participatory creative activities
Ritualization of homologous emotional expression	Sacredness	Immersive review of anime Viewing anime pilgrimage sites with a mindset of reverence Feeling a psychological connection with sacred anime locations Experiencing the emotions and values of anime characters Sharing a collective emotional landmark with the community
	Ritualization	Standardized actions and postures Precise replication down to the smallest details Maximizing the restoration of anime scenes Strict adherence to costumes, props, perspectives, composition, or lighting
The hierarchical construction of shared emotional identity	Aesthetic identification	Composition and color schemes Character dynamics Lighting aesthetics Overall environmental ambiance Color aesthetics
	Cultural identification	Linking local scenery and customs to the life journey of characters Associating character lifestyles with Japanese culture Connecting character principles with positive values Relating anime scenes to aesthetic appreciation
	Identity identification	Sustained emotional engagement with anime communities and collective interactions Developing character preferences to express emotional attitudes Actively participating in collective anime pilgrimage activities Holding a positive attitude toward anime pilgrimage as a whole Continuously following news about anime and its associated sacred sites
Community-based viral spread of shared anime fandom	Emotional investment	Core community leaders create high-quality anime-related content out of passion The increase in outstanding creators leads to the expansion of anime communities that share emotions More anime enthusiasts participate in collective emotional expression within these communities Anime communities sharing emotions become increasingly networked
	Content creation	Members of anime communities engage in secondary creations based on real-life locations Secondary creations generate emotional resonance Specific symbols gain symbolic meaning Group identifiers gradually emerge More anime community members visit real-life locations for pilgrimages
	Engagement expansion	Offline experiences enhance or update online community guides Big data algorithms further target and promote to specific demographics The participation rate in anime communities rises
Dynamic mechanisms of shared emotional influence	Anime type	Healing/slice-of-life works are more likely to evoke deep empathy Action/combat works trigger visitors' imitation impulses and community identity School romance enthusiasts tend toward romantic check-ins and emotionally commemorative tourism Sci-fi enthusiasts exhibit a behavior pattern of technological curiosity and urban exploration Fantasy adventure works inspire exploratory travel motivations Historical/cultural works evoke knowledge-seeking tourism Music/idol works drive limited-time event economies
	Cultural distance	Share the east Asian cultural sphere Relatively familiar with Japanese culture Less understanding of Shinto and shrine culture Limited grasp of mono no aware (the beauty of transience) Difficulty comprehending the spirit conflicting ideals of “The chrysanthemum and the sword”
	Experience quality	Authenticity of anime scenes Interactive participation in anime-themed activities Professionalism of anime experience services Degree of emotional embodiment in anime Realistic portrayal of anime characters
	Official administration	Relatively strict travel policies to japan Restrictions on the number of tourists in popular scenic spots Attracting talent and visitors through the “Cool Japan” Cultural policy Diverse anime marketing strategies

#### 4.3.1 Phase 1: symbolization of homologous emotions

Anime tourists traveling to Japan progressively internalize virtual symbols from anime into personal emotional vessels through a symbolic construct of “visual symbol recognition—cultural meaning decoding—personalized meaning reconstruction.” This establishes a profound connection between reality and fiction. Their emotions gradually become symbolized across three developmental stages: perception, cognition, and internalization.

In emotional perception stage, the emotion is anchored by visual symbols and initiates a preliminary link between the virtual and real worlds. Anime tourists traveling to Japan first identify iconic scenes in anime, forming prescribed symbolic visual features. For example, the anime tourists traveling to Japan recognize the circular outline of Lake Suwa in Your Name, or spotting the seaside railway tracks near Kamakura Koko-mae Station from Slam Dunk. These observations activate symbolic memories of the anime in their minds.

In the emotional cognition stage, anime tourists traveling to Japan attempt to interpret the symbol from a complex network of meanings. As a result, they transform ordinary geographical spaces into real-world carriers of symbolic anime scenes, using physical media to strengthen the relationship between anime scenes and real scenes, thereby connecting the anime world with the real world. For example, when anime tourists traveling to Japan learn about the connection between the shrine scene in “Your Name.” and Japanese Shinto culture, they view the shrine scene as an anchor point of the anime world in reality, and by studying the unique settings of the anime and imitating the characters, they imbue this physical space with emotional and cultural connotations, transforming the scene into a cultural symbol capable of carrying the characters' emotions and worldview.

In the emotional internalization stage, anime symbols are primarily transformed into unique carriers in personal memory through creative practices. At this point, Japanese tourists are no longer satisfied with passive perception. Instead, they actively participate in and engage in creative activities, integrating anime symbols into their practical actions, such as filming fan-made short videos, writing pilgrimage travelogues, and designing exclusive route maps. These creative practices allow the symbols to transcend the work itself and become unique identifiers that carry personal emotions and memories.

#### 4.3.2 Phase 2: ritualization of homologous emotional expression

Anime tourists traveling to Japan project their emotions onto physical spaces through ritualistic behaviors characterized by sanctity and procedural scene reenactments, constructing emotional landmarks that embody both individual uniqueness and community commonality, thereby manifesting the meaning of the virtual world in reality.

The ritualization of emotional expression manifests in the replication of standard actions. Anime tourists traveling to Japan often reproduce anime scenes with an almost “pilgrimage-like” attitude, meticulously adhering to the original perspectives, compositions, and even lighting conditions when taking photos. A comparison of real-world anime pilgrimage sites can be found in Table 6.

**Table 6:** Comparison of anime pilgrimage sites and real locations.

**Pilgrimage comparison**	**Slam dunk**	**NANA**	**Cardcaptor Sakura**
Similarities to anime scenes	Pose, time of day, camera angle	Time of day, camera angle, direction of tourist's gaze	Background setting, camera angle, tourist's standing position
Differences from anime scenes	Tourist attire, color tone of the image	Store signage, tourist attire	Tourist pose, color tone of the image
Characteristics of the scene in the anime	The anime scenario features a lowered railway crossing gate, tracks below for passing trains, and a corresponding red signal light indicating that pedestrians are prohibited from crossing. On either side of the gate, there is a male anime character and three female anime characters. The three female characters are chatting cheerfully, facing the viewer. The male character stands quietly gazing at the three female characters, with his back to the viewer, holding a black bag on his right shoulder with his right hand.	The anime scene is set in front of a building at night. The building is brightly lit and has glass doors. The protagonist is riding an electric scooter, wearing a helmet, and the scooter's headlight is illuminated. In the background, there are two pedestrians.	The anime depicts a group of people watching jellyfish. There are numerous jellyfish, and they are glowing, set against a blue background. Among the crowd, a man and a woman stand in the center, with an adult and a child on the left, and a couple on the right. There is some space between these three groups.
Real-life location image	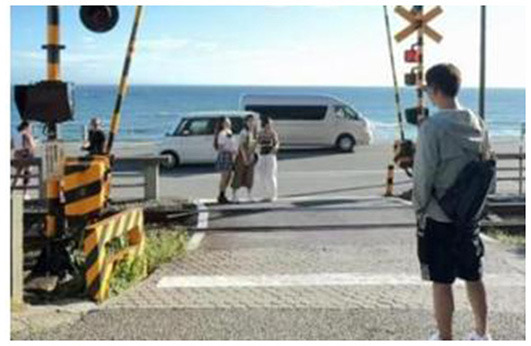 Location: Beach (Kanagawa) Source: Taken by the author	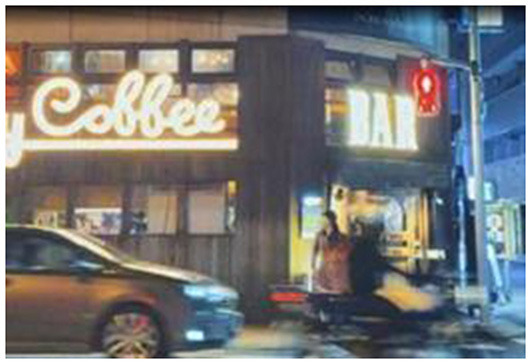 Location: Jackson Hole (Chōfu, Tokyo) Source: Taken by the author	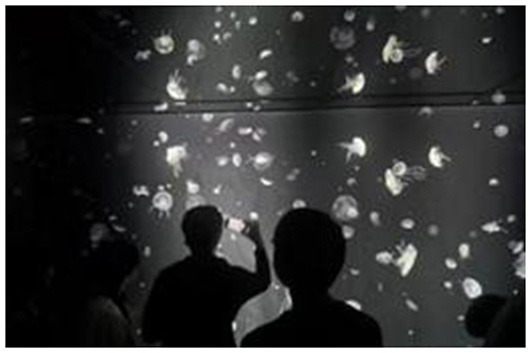 Location: Osaka Aquarium Kaiyukan (Osaka) Source: Taken by the author

The first image is at the Beach of Kamakura High School, a real-world location from the anime Slam Dunk. Anime tourists precisely replicate the classic poses, such as carrying a backpack and striking the same angle for their check-in photos. In front of the camera, three other girls can also be seen mimicking the anime characters' movements. Strangers form homologous emotional bonds through their shared reenactments of the same anime scene. This behavior transforms physical spaces, such as railroad tracks and seashores, into emotional landmarks of collective memory through bodily practices, reinforcing the symbolic and emotional association.

The second image in Table 6 captures a moment in Jackson Hole, where a tourist captures a speeding motorcycle at a similar time-point as seen in NANA. A woman in the distance gazes just like the anime protagonist, resonating emotionally through this ritualized reenactment.

The third image in Table 6 reproduces a scene from Cardcaptor Sakura (Episode 2: “Aquarium”), where Sakura Kinomoto and Tomoyo Daidouji observe jellyfish. Although the pilgrim's photo appears in grayscale—possibly due to personal interpretation—the camera angle and positioning closely match the anime frame.

These scene reenactments construct a “sacralized” emotional loop, enabling anime tourists to reconstruct the meaning of the virtual world in real space. Ultimately, this phase form a cultural identity system that blends individual uniqueness with collective recognition.

#### 4.3.3 Phase 3: the hierarchical construction of shared emotional identity

Shared emotional identity follows a hierarchical structure of “aesthetic identity—cultural identity—identity recognition.” It strengthens group belonging through exclusive symbolic interactions, ultimately forming a step-by-step identity construction system.

The foundational level is aesthetic identity. When anime tourists traveling to Japan first encounter an anime, they are immediately struck by visual elements such as the composition, color schemes, and character movements, forming an addictive emotional connection at a sensory level. For example, in The Girl Who Leapt Through Time, the dappled sunlight filtering through leaves and the dynamic wrinkles of a character's skirt while running create aesthetic expectations for the filming location, Sendagaya Nishien Garden.

Cultural identity constitutes the middle layer. Tourists develop cultural connections by understanding the symbolic meanings within the works. For instance, through “anime pilgrimage” they associate local specialties with anime characters' life journeys, forming shared cultural understandings like “going to Kamakura means experiencing Sakuragi Hanamichi's youth.” This transforms real locations into tangible representations of anime culture, linking fictional symbols to real-world cultural practices.

The highest level is identity recognition. Tourists establish long-term identity bonds through sustained emotional investment and participation in gatherings like anime conventions and pilgrimages. For example, when the final season of Attack on Titan aired, fans collectively mourned at the Isayama Hajime memorial stone in Tokyo. On the other hand, this identity based on shared emotional connections can also be exclusive—Evangelion fans may divide into factions like Rei Ayanami supporters and Asuka Langley supporters, reinforcing subgroup identities by favoring different characters. However, overall, this identity remains inclusive, allowing anime tourists traveling to Japan to quickly form emotional bonds on a broader scale.

#### 4.3.4 Phase 4: community-based viral spread of shared anime fandom

Shared emotional connections in anime fandom achieve viral spread through core community leadership, driven by social interactions and algorithm recommendations. This dissemination forms a content production loop of “emotional engagement → content creation → participant expansion.” The spread occurs through three key steps.

First, core community leaders produce high-quality, professional anime-related contents such as travel guides and in-depth analyses. Motivated by their passion for anime, these creators generate premium content that shapes the perspectives of casual fans. As more skilled anime travel creators join, the shared emotional community grows.

Second, community members experience emotional resonance through second creations (fanworks) based on real anime locations, forming collective memories. During this process, specific symbols gain symbolic meaning and become group identifiers. Inspired by these shared experiences, fans travel to Japan and engage in offline activities that reinforce these emotional bonds and symbolic representations.

Lastly, after completing their anime pilgrimages, tourists typically share their offline experiences in online communities. This step fulfills their need for emotional validation, identity recognition, and value reinforcement. Social media algorithms then amplify this emotional spread by recommending personalized content, increasing user engagement.

Through these three phases, shared emotional connections create a closed-loop “emotional economy.” Ultimately, anime tourists traveling to Japan and creators accelerate this emotional spread through both online and offline interactions, achieving both cultural and economic value growth.

#### 4.3.5 Phase 5: dynamic mechanisms of shared emotional influence

The intensity of shared emotional connections in anime tourists traveling to Japan is moderated by multiple factors including media genre, cultural distance, and scene authenticity.

The moderating mechanism of emotional type stems from differences in anime's emotional tones. During pilgrimage tours, anime tourists often experience: a pilgrimage-like longing for 2D nostalgia, surreal overlap between real and fictional scenes, and a sense of belonging among fellow fans. Different anime genres also significantly shape tourist emotions—for example, healing-themed works (e.g., Natsume's Book of Friends) foster deeper emotional resonance, while action-packed series (e.g., Demon Slayer) trigger stronger behavioral motivation.

Cultural distance as a moderating factor depends on tourists' prior knowledge of Japanese culture. This directly impacts their ability to decode cultural symbols and form emotional connections. For instance, without basic understanding of Shinto traditions, tourists may only perceive shrine scenes superficially, failing to grasp their cultural context. Even within the same East Asian cultural sphere, the customs and traditions of different countries can be both an attraction and a barrier. Therefore, it is essential to handle them appropriately to allow tourists to experience authentic culture.

Experience quality encompasses multidimensional elements like scene authenticity, interactive engagement, and professional services, all crucial for emotional reinforcement. High-quality experiences enhance immersion and emotional attachment—for example, Demon Slayer: Mugen Train's 1:1 replica train cabin allowed fans to sync breathing patterns with Kyojuro Rengoku, while live voice acting deepened immersion. Such encounters amplified emotional investment, making character deaths more impactful.

Notably, these moderating mechanisms dynamically evolve during trips. First-time tourists initially explore locations out of curiosity. Gradually, under these influences, surface-level aesthetic appreciation transforms into deeper cultural engagement, reflecting a staged progression from visual fascination to spiritual belonging.

**Figure 2:**
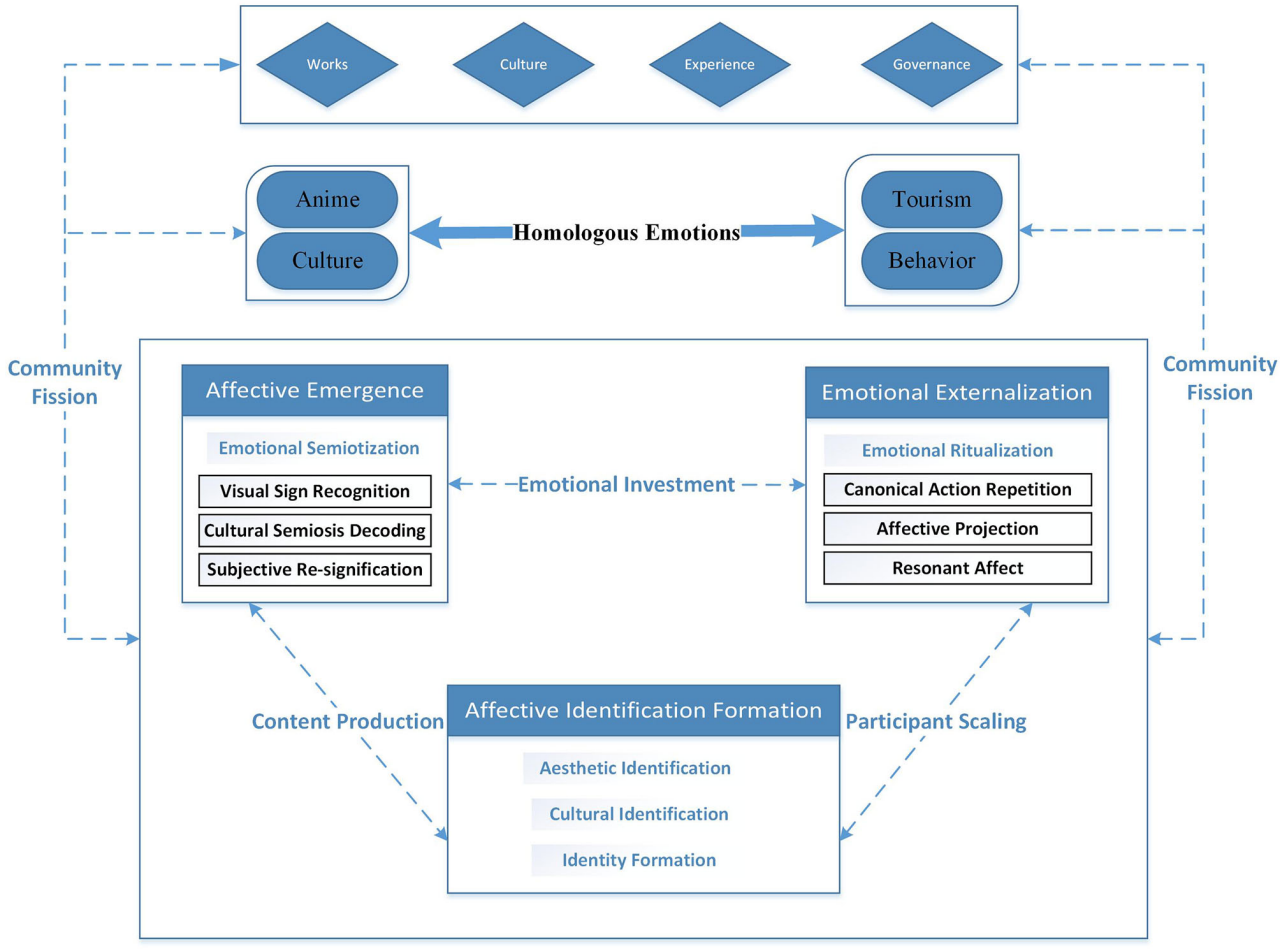
The formation mechanism of homologous emotions among anime tourists traveling to Japan.

Overall, the formation mechanism of homologous emotions among anime tourists traveling to Japan is illustrated in Figure 2.

### 4.4 Marketing strategy analysis

With the rapid expansion of anime tourists traveling to Japan, effectively leveraging anime fans' pilgrimage behavior and homologous emotions to drive tourism consumption has become crucial. Based on prior research findings, this paper proposes four marketing strategies to deepen the experience of anime tourists and amplify market influence through social media.

#### 4.4.1 Ritualized construction strategy for cultural symbols

The core value of anime tourism lies in the emotional connection between the virtual and the real. Therefore, a ritualized marketing mechanism based on a cultural symbolic system should be established. Marketing efforts must systematically transform the visual symbols, narrative elements, and philosophical connotations within anime works into tangible and participatory experiential activities. First, specific scenes must be imbued with sacred cultural meaning, followed by standardized behavioral norms to reinforce the sense of ritual, ultimately forming community practices that serve identity recognition. At the operational level, excessive commercialization that dilutes symbolic meaning should be avoided to preserve the integrity and depth of the original aesthetic.

#### 4.4.2 Spatiotemporal encoding mechanism for emotional value

Anime tourism consumption exhibits significant temporal and spatial dependencies. From a time-based perspective, marketers should leverage the transient nature of ^*^mono no aware^*^ (the pathos of things) to create scarcity through limited-time offerings and seasonal cycles. In terms of spatial dimensions, physical locations must overlap with narrative spaces, transforming real-world places into emotional vessels that carry collective memory. Throughout implementation, homogenization must be guarded against to maintain value tension across all levels.

#### 4.4.3 Cultivation of an industrial collaborative ecosystem

Maximizing the economic benefits of anime tourism depends on constructing a cross-industry value network. Institutional innovations are needed to break down barriers between content production, spatial operations, and service provision, forming a synergistic ecosystem centered on intellectual property (IP), connected through experiences, and supported by services. In fostering this ecosystem, it is crucial to preserve the creative autonomy of all involved entities, prevent excessive standardization that leads to homogenized experiences, and maintain market dynamism and diversity.

#### 4.4.4 Participatory production in digital dissemination

The integration of social media has transformed anime tourism from a one-way consumption model into a multi-directional co-creation process. A trinity digital communication system—comprising “platform empowerment, community incentivization, and algorithmic optimization”—must be established. This system integrates tourists' creative labor into the value production process, forming a positive cycle of “emotional engagement, content output, and amplified influence.” During operation, the autonomous nature of subcultural communities must be safeguarded, striking a balance between commercial promotion and cultural purity while preventing algorithmic logic from eroding community ecosystems.

## 5 Discussion

This study on pilgrimage behavior and homologous emotions in anime tourism provides important additions to existing theories such as tourist symbolic consumption, emotional geography, and the tourist gaze. We found that anime tourists' interactions with destinations are no longer one-way or passive observation processes, but rather highly interactive and immersive experiences. These experiences possess dual characteristics: they include both visual capture of real scenes and emotional projection of virtual symbols. As one interviewee at a filming location from “Your Name.” described: “When I stood on the steps where the protagonists met in the film, those classic scenes automatically played in my mind, as if I had crossed into a two-dimensional world.” While this phenomenon was noted in Macionis' (2004) research on film tourism, our study found that the unique “dimensional crossover” sensation in anime tourism is stronger and more concrete.

Regarding tourist behavior, cultural tourism practices are increasingly shifting from reliance on cultural carriers to dependence on digital media. Our findings offer new insights into Graburn's (1983) “tourism ritual theory.” We discovered that anime tourism is essentially a “mediated ritual,” where the sense of ritual comes not only from physical movement through space but also relies on digital performances in the mobile internet era. Compared to traditional tourism memorial behaviors (such as taking photos), contemporary anime tourists pay more attention to sharing carefully selected and curated content on social platforms. This “digital authentication” process has become an indispensable part of the pilgrimage experience. Data from the Japan Tourism Agency in 2022 shows that 87% of anime tourists post at least three pilgrimage-related contents on social media, and 62% adjust their itineraries based on content shared by others. Although Reijnders' (2011) research on media tourism touched upon this phenomenon, it did not fully recognize the reconstruction effect of digital platforms on the entire experience process.

Compared with existing cultural tourism research, this study reveals new identification mechanisms in anime communities during the digital era. First, the decoding of anime scene symbols shows dual permeability characteristics. Anime tourists traveling to Japan imbue real scenes with virtual symbolic meanings, making real elements anime-like. This two-way interaction confirms the expanded applicability of Appadurai's “mediascape” theory in new consumption contexts. Second, ritualized practices create emotional reinforcement effects. The exact recreation of scenes down to camera angles is essentially a modern interpretation of Cohen's “sacred sense of tourism.” Such behavior also forms a “tacit community” among unfamiliar tourists, reflecting the strong regulating power of virtual emotions on real-world behaviors. Third, community communication builds emotional value chains. Anime core fans create pilgrimage guides through a “creation-imitation-recreation” cycle, ultimately generating emotionally valuable assets. The production efficiency of this content correlates with fans' emotional investment, providing empirical support for “emotional economy” theory.

In terms of local cultural development and marketing, our research offers new perspectives on Gospodini's (2004) “iconic architecture” theory. We found that the landmark status of anime filming locations does not stem from architectural aesthetic value, but rather from emotional significance attached through media narratives. Therefore, cultural tourism carriers and narratives are equally important. In terms of specific marketing content, anime culture differs from traditional national imagination. Anime tourists traveling to Japan construct a trans-border emotional identification based on specific cultural symbols through pilgrimage. Thus, cultural tourism narratives should start locally and go global. With the globalization of culture, local cultural identity has broken through subcultural boundaries to become mainstream consumer practice. In terms of specific marketing models, each strategy requires balancing commercial goals with authentic emotional connections to foster sustainable anime tourism growth. Japan's successful experience lies in establishing a “win-win-win” business model: copyright holders obtain revenue through licensing, local governments gain tourism income, and tourists receive in-depth experiences. This approach of comprehensively injecting IP value into destinations can create more lasting economic benefits than simply developing derivative products.

This study has two methodological limitations: firstly, the sample mainly consists of tourists from East Asian cultural circles, failing to fully examine the moderating effect of cultural distance; secondly, it did not track the long-term attenuation patterns of homologous emotions. Follow-up research could use tracking methods to compare the emotional persistence differences between “pilgrimage-type” and “check-in-type” tourists, which would provide important references for managing the lifecycle of anime tourism products.

## 6 Conclusions

This study systematically examines the pilgrimage behavior and homologous emotions phenomenon among Chinese anime tourists traveling to Japan. The research shows that the pilgrimage behavior of Chinese anime tourists traveling to Japan is essentially a cultural practice that connects the virtual and the real through mediated rituals. The study reveals three key findings. First, in terms of behavioral characteristics, anime tourists construct a unique “pilgrimage grammar” through symbolic consumption (68% purchase rate of limited-edition goods), productive content creation (87% social media posting rate), and ritual sanctity (92% accuracy rate in scene recreation and composition matching). Second, the homologous emotion mechanism displays dynamic and structured features. Emotion formation follows a “five-stage model,” starting from symbolic decoding, undergoing ritual reinforcement, hierarchical identity formation, community dissemination, and ultimately being moderated by multiple factors such as experience depth and cultural decoding ability. Third, the study proposes marketing strategies including ritualized construction strategy for cultural symbols, spatiotemporal encoding mechanism for emotional value, cultivation of an industrial collaborative ecosystem, participatory production in digital dissemination. Each marketing strategy needs to balance cultural authenticity with commercial feasibility. In summary, theoretically, this study expands the conceptual scope of tourism research and reveals trends in cultural consumption transformation in the digital era. Practically, it identifies pathways to connect contemporary emotional resonance with cultural needs, laying a research foundation for the innovative development of the cultural and tourism industries.

## Data Availability

The original contributions presented in the study are included in the article/supplementary material, further inquiries can be directed to the corresponding author.

## Appendix

**Table A1:** Results of exploratory factor analysis.

**Factor**	**Measurement items**	**Factor loadings**				**Reliability (coefficient)**
		**F1**	**F 2**	**F3**	**F4**	
Daily anime perception	Main channels for accessing Japanese anime	0.795				0.708
	Time spent weekly watching/reading Japanese anime	0.689				
	Favorite types of Japanese anime	0.694				
	Frequency of participating in anime-related activities	0.648				
Anime tourism behavior	Main motivations for traveling to Japan		0.820			0.635
	Main reasons for choosing anime-related destinations		0.707			
	Main areas of consumption in Japan		0.704			
	Whether to visit anime filming locations for “sacred site pilgrimage”		0.689			
	Main behaviors during “sacred site pilgrimage”		0.791			
Emotional experience evaluation	Main sources of anime's appeal			0.819		0.643
	Emotional experiences most desired from anime tourism			0.701		
	Degree of influence of specific “sacred site pilgrimage” behaviors on emotional experience			0.664		
Social media usage	Platforms for sharing daily anime tourism experiences				0.730	0.687
	Types of content shared about daily anime tourism				0.714	
	Degree of influence of social media on anime tourism decisions				0.739	
